# *Cryptosporidium hominis* Infection of the Human Respiratory Tract

**DOI:** 10.3201/eid1303.060394

**Published:** 2007-03

**Authors:** Rubén Mercado, Gregory A. Buck, Patricio A. Manque, Luiz Shozo Ozaki

**Affiliations:** * Facultad de Medicina, Universidad de Chile, Santiago, Chile; † Virginia Commonwealth University, Richmond, Virginia, USA

**Keywords:** Cryptosporidium hominis, Cryptosporidium parvum, pulmonary cryptosporidiosis, respiratory tract, HIV, 18S rRNA, PCR, dispatch, Suggested Citation for this Article: Mercado R, Buck GA, Manque PA, Ozaki LS. *Cryptosporidium hominis* infection of the human respiratory tract. Emerg Infect Dis [serial on the Internet]. 2007 Mar [*date cited*]. Available from http://www.cdc.gov/EID/content/13/3/462.htm

## Abstract

*Cryptosporidium* oocysts, observed in a natural sputum sample of a patient with HIV, were further studied by using DNA markers to determine the species of the parasite. *C. hominis* was identified as the species infecting the patient’s respiratory tract, a finding that strengthens evidence regarding this pathogen’s role in human disease.

Intestinal cryptosporidiosis is a common parasitic disease that causes self-limiting diarrhea in immunocompetent persons (*1*). Higher frequencies of *Cryptosporidium* infection are observed in immunocompromised humans, and the main clinical pattern of the infection in these persons is a chronic, life-threatening secretory diarrhea (*2*).

At least 8 species of *Cryptosporidium* are described as infecting humans. *C. hominis* and *C. parvum* are the most frequently observed in intestinal infections in humans (*3*). *C. meleagridis* is also detected both in immunocompetent and immunodeficient patients, although at a lower rate than *C. parvum* (*4*).

Respiratory tract infection by *Cryptosporidium* spp. has been described for immunodeficient persons, most all of whom were coinfected with HIV. However, pulmonary cryptosporidiosis was also described in patients without HIV infection (*5**,**6*). In all cases, no systematic identification of the species of *Cryptosporidium* was pursued except by Meamar et al. (*7*), in which the parasite was identified as *C. parvum*.

We describe the detection and identification of *C. hominis* in the respiratory secretions of a patient with HIV (sample Chile01). We used an oligonucleotide species-specific method and sequencing of parts of the 18S rRNA gene to determine the species of *Cryptosporidium***.** Both analyses showed that the species of *Cryptosporidium* present in the pulmonary secretion of this patient was *C. hominis*.

## The Study

**Figure 1:**
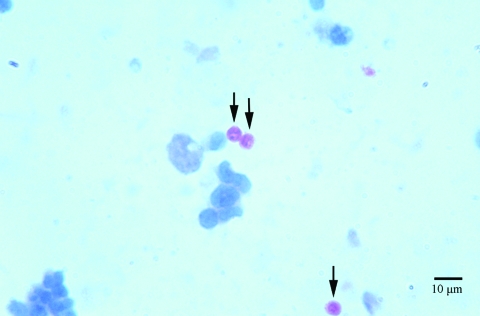
Oocysts (arrows), stained by using the Ziehl-Neelsen method, in sputum from the patient with HIV.

In September 2004, a 58-year-old man, who received an HIV diagnosis in 1996, was hospitalized with respiratory symptoms characterized by persistent cough. *Cryptosporidium* oocysts were detected in a sputum sample from the patient by using Ziehl-Neelsen stain (Figure 1). An aliquot of ≈10 mL of respiratory secretion was obtained. DNA was extracted as follows: 200 μL fluid was centrifuged and the pelleted material digested overnight at 65ºC with proteinase K in the presence of 10% sodium dodecyl sulfate. We then sequentially extracted the digested material with phenol, phenol-chloroform-isoamyl alcohol, and chloroform-isoamyl alcohol. The DNA were then precipitated with sodium acetate and ethanol, and after centrifugation, the pelleted DNA was dissolved in 50 µL of water.

**Figure 2:**
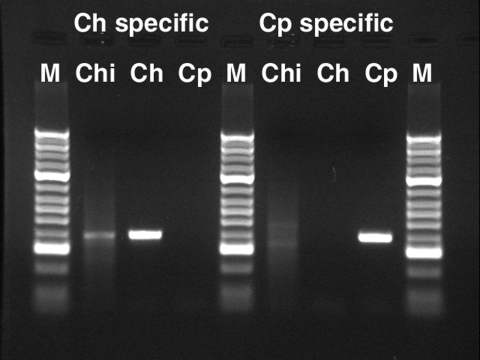
Agarose gel electrophoresis of DNA fragments amplified with *Cryptosporidium* species–specific Lib13 primers (*7*). Ch, *C. hominis*; Cp, *C. parvum;* M, DNA molecular marker (Bioline, Randolph, MA, USA; HyperLadder II, higher intensity bands: 0.3, 1, and 2 Kbp); Chi, sample Chile01.

For molecular typing, we first used the species-specific oligonucleotide PCR assay Lib13, as described by Tanriverdi et al. (*8*), with a new sense oligonucleotide primer based on the genome sequences of *C. hominis* (*9*) and *C. parvum* (*10*). The new primer, Lib13SF02 (5′-TTTTTTCATTAGCTCGCTTC-3′), a fragment of ≈400 bp, was amplified specifically from *C. hominis* DNA with the anti-sense primers Lib13SRT-1 (5′-ATTTATTAATTTATCTCTTACTT-3′) and from *C. parvum* DNA with Lib13SRT-2 (5′-ATTTATTAATTTATCTCTTCG-3′) (Figure 2). Amplifications were carried out in a PCR mixture of 10 μL containing 0.25 mmol/L of each dNTP, 300 pmol/L of each olignucleotide, and 1 unit of Taq DNA polymerase (HotMaster, Eppendorf, New York, NY, USA). Temperature cycling was performed on a GeneAmp PCR System (ABI, Foster City, CA, USA) with initial denaturation performed at 95ºC for 5 min, then 40 cycles at 95ºC for 30 s, 52ºC for 30 s, and 68ºC for 30 s. The mixture was then cooled to 4ºC.

The region from bases 7 to 1036 (numbering according to *C. hominis* sequence GenBank no. L16996) of the 18S rRNA gene was sequenced from DNA fragments amplified using the primers 18SF (5′-GTTGATCCTGCCAGTAGTC-3′) and 18SR (5′-TAAGGTGCTGAAGGAGTAAGG-3′) and cloned into the TOPO TA vector (Invitrogen, Brandford, CT, USA) by using standard techniques. Automated sequencing was performed directly on the amplified fragments or on cloned fragments at the Nucleic Acid Research Facilities of Virginia Commonwealth University. All DNA sequences were analyzed by using Sequencher (Gene Codes Co., Ann Arbor, MI, USA).

Figure 1 shows fuchsia (Ziehl-Neelsen)–stained sputum from the patient. Three *Cryptosporidium* oocysts (arrows) with typical sizes ≈5 μm in diameter are visible. Measurements were performed in a calibrated microscope as described by Mercado and Santander (*11*).

A Lib13 PCR assay (*8*) was performed on the DNA purified from the respiratory secretion material, and the results are shown in Figure 2. With the *C. hominis* specific-primer pair (LIBF02/Lib13SRT1), a fragment of the expected size (≈400 bp) was amplified with the Chile01 isolate DNA (Figure 2, lane 2). With this primer pair, we also obtained amplification with the *C. hominis* isolate TU502 (*12*) DNA (Figure 2, lane 3) but not with the *C. parvum* isolate Moredun (*13*) DNA (Figure 2, lane 4). Conversely, the *C. parvum*–specific primer pair (LIBF02/Lib13SRT2) amplified a fragment only with the *C. parvum* DNA (Figure 2, lane 8). No amplification was observed with the Chile01 (Figure 2, lane 6) or the *C. hominis* DNA (Figure 2, lane 7). DNA sequencing of the amplified fragments confirmed the polymorphism to be that of *C. hominis* (results not shown).

We also analyzed the 18S small subunit rRNA gene by amplifying and sequencing an 18S rRNA fragment from the sputum DNA. Amplification and sequencing was concomitantly performed with the *C. hominis* and *C. parvum* DNA. A polymorphic site exists in *C. hominis* as a stretch of 10 to 12 thymines (T_10–12_), while in *C. parvum,* the sequence is TA:::TATATTTT (*146*). The 18S rRNA polymorphic sequence found in the sputum sample DNA (GenBank accession no. DQ286403) is that of *C. hominis*, with a stretch of 11 Ts (Table). Few other nucleotide polymorphisms were found between the sequences (results not shown), which reflect intraisolate variations (*14*). The results with Lib13 assay and the partial 18S rRNA sequence analysis, therefore, identify the species of *Cryptosporidium* infecting the respiratory tract of this patient as *C. hominis*.

**Table:** Nucleotide sequences at the T polymorphic site of the 18S rRNA of the pulmonary sample of *Cryptosporidium* (Chile01), *C. parvum* (Cp), and *C. hominis* (Ch)

Isolate	18S rRNA sequence at 670–710
Chile01	5′-CATAATTCATATTACTATTTTTTTTTTTAGTATATGAAATT-3′
Cp	5′-CATAATTCATATTACTA:::TATATTTTAGTATATGAAATT-3′
Ch	5′-CATAATTCATATTACTA:TTTTTTTTTTAGTATATGAAATT-3′

## Conclusions

Human cryptosporidiosis is better known as an intestinal disease both in immunocompetent and immunocompromised persons. Little information exists, however, on human pulmonary disease caused by *Cryptosporidium* spp., which reflects either the low prevalence of pulmonary cryptosporidiosis or the lack of testing in immunocompetent hosts. Further, if performed, current diagnostic tests may not be sensitive enough to detect the parasite.

In immunocompetent children with intestinal cryptosporidiosis, respiratory symptoms have been noted more frequently than expected (*5*). Studies are needed in immunocompetent persons, especially children <2 years of age, who belong to a group at high risk for intestinal, and by extension pulmonary, cryptosporidiosis.

More information about which species of this pathogen infect humans and the pathogenic patterns each species produces is needed. As we determined here, *C. hominis* have the capacity to adapt to different physiologic environments, such as intestinal and respiratory tract tissues. Our findings provide additional evidence supporting the role of this species of *Cryptosporidium* as a human pathogen and the need to evaluate the importance of pulmonary cryptosporidiosis as a disease in the immunocompromised host.
